# Supplementary material for: Association between Sarcopenia, Falls, and Cognitive Impairment in Older People: A Systematic Review with Meta-Analysis
Source: Int J Environ Res Public Health. 2023 Feb 25;20(5):4156. doi: 10.3390/ijerph20054156 (PMC10002412; doi:10.3390/ijerph20054156)
Supplement: Supplementary file 1 [file ijerph-20-04156-s001.zip › ijerph-2197819-supplementary.pdf]

International Journal of
Environmental Research
and Public Health

Table S1. First search.

CINAHL

Nº	Estrategy	Results
1	MH "Accidental Falls"	25.583
2	Accidental Falls	25.700
3	Accidental Fall	25.700
4	1 OR 2 OR 3	25.682
5	MH "Aged"	896.302
6	Aged	1.078.827
7	5 OR 6	1.078.827
8	MH "Sarcopenia"	3.480
9	Sarcopenia	5.468
10	8 OR 9	5.468
11	MH “Cognition Disorders”	33.083
12	Cognitive Disorders	27.610
13	Cognitive Dysfunction	26.553
14	MH "Cognitive Aging"	380
15	Cognitive Aging	3.323
16	11 OR 12 OR 13 OR 14 OR 15	37.387
17	4 AND 7 AND 10 AND 16	11

PUBMED (MEDLINE)

Nº	Estrategy	Results
1	"Accidental Falls"[Mesh]	27.571
2	Accidental Falls	28.376
3	Accidental Fall	28.784
4	1 OR 2 OR 3	28.289
5	"Aged"[Mesh]	3.413.774
6	Aged	5.863.081
7	5 OR 6	5.863.081
8	"Sarcopenia"[Mesh]	7.919
9	Sarcopenia	15.625
10	8 OR 9	15.625
11	"Cognitive Dysfunction"[Mesh]	31.863
12	Cognitive Dysfunction	66.208
13	11 OR 12	46.261
14	4 AND 7 AND 10 AND 13	7

Table S2. Studies excluded from this review.

No.	TITLE	AUTHORS	YEAR	REASON
1	Cognitive and behavioral factors associated to probable sarcopenia in community-dwelling older adults	Souza LF, Fontanela LC, Gonçalves C, Mendrano AL, Freitas MA, Danielewicz AL, Avelar NCP.	2022	There is no investigation on the occurrence of falls
2	Combined impact of positive screen for sarcopenia and frailty on physical function, cognition and nutrition in the community dwelling older adult	Lee HX, Yeo A, Tan CN, Yew S, Tay L, Ding YY, Lim WS.	2021	It was not conducted exclusively with elderly people and excludes participants with cognitive impairment
3	Prevalence and associates of fear of falling among community-dwelling older adults	Bahatöztürk G, Kiliç C, Bozkurt ME, Karan MA.	2021	Elderly people with cognitive impairment have been excluded
4	Possible sarcopenia and impact of dual-task exercise on gait speed, handgrip strength, falls, and perceived health	Merchant RA, Chan YH, Hui RJY, Lim JY, Kwek SC, Seetharaman SK, Au LSY, Morley JEB.	2021	It excludes elderly people with low mental status examination scores or with a known diagnosis of dementia or<br>cognitive impairment
5	The Cumulative Impact of Sarcopenia, Frailty, Malnutrition, and Cachexia on Other Geriatric Syndromes in Hospitalized Elderly	Rasheedy D, El-Kawaly WH	2021	It does not provide statistical analysis results regarding the association between sarcopenia and falls. In addition, it does not show the results regarding the occurrence of falls.
6	Fatores associados a quedas em idosos com e sem déficit cognitivo acolhidos em serviço de atendimento especializado de geriatria	Abreu AMS	2021	There is no diagnosis of sarcopenia
7	Prevalência de sarcopenia em idosos de um município do nordeste brasileiro	Rodrigues AAGS	2020	The study excludes elderly people with cognitive impairment
8	Relationship Between Fear of Falling, Fear-Related Activity Restriction, Frailty, and Sarcopenia	Merchant RA, Chen MZ, Wong BLL, Ng SE, Lim JY.	2020	Elderly people with severe cognitive disorders, dementia, and cognitive impairment were excluded

9	The Quick Physical Activity Rating (QPAR) scale: A brief assessment of physical activity in older adults with and without cognitive impairment	Galvin JE, Tolea MI, Rosenfeld A, Chrisphonte S.	2020	It does not investigate the occurrence of previous falls
10	Sarcopenia e quedas em idosos atendidos em uma clínica escola	Borges CL.	2019	The study has as exclusion criteria elderly people with cognitive impairment
11	Os fatores clínicos e físico-funcionais predizem quedas em idosos com déficit cognitivo?	Araújo RS, Nascimento ER, Barros RS, Ritter SRF, Abreu AMS, Garcia PA.	2019	There is no diagnosis of sarcopenia
12	Prevalence and Associated Factors of Sarcopenia and Frailty in Parkinson's Disease: A Cross-Sectional Study	Peball M, Mahlknecht P, Werkmann M, Marini K, Murr F, Herzmann HH, et al.	2019	There is no research on the association between sarcopenia and falls in the cognitively impaired elderly
13	Alterações cognitivas e funcionais e sua relação com o risco de quedas em idosos da atenção primária	Ferreira Júnior TC	2018	It does not investigate the occurrence of falls
14	Balance problems and fall risks in the elderly	Cuevas-Trisan R	2017	Secondary study
15	Relación entre los subdominios de la función ejecutiva y balance postural: implicancias en el riesgo de caídas en el adulto mayor autovalente residente en la comunidad	Cid NFM, Navarrete, Martínez CCA	2017	It excludes cognitively impaired elderly people and there is evaluation of sarcopenia and cognitive impairment
16	Quality of life and physical components linked to sarcopenia: The SarcoPhAge study	Beaudart C, Reginster JY, Petermans J, Gillain S, Quabron A, Locquet M, et al.	2015	It does not evaluate the occurrence of falls and the cognitive impairment is not evidenced in the results
